# Why do female mice mate with multiple males?

**DOI:** 10.1007/s00265-013-1604-8

**Published:** 2013-08-09

**Authors:** Kerstin E. Thonhauser, Shirley Raveh, Attila Hettyey, Helmut Beissmann, Dustin J. Penn

**Affiliations:** 1 Konrad Lorenz Institute of Ethology, Department of Integrative Biology and Evolution, University of Veterinary Medicine, Vienna, Savoyenstraße 1a, 1160 Vienna, Austria; 2 Department of Environmental Sciences, Zoology and Evolution, University of Basel, Vesalgasse 1, 4051 Basel, Switzerland; 3 “Lendület” Evolutionary Ecology Group, Plant Protection Institute, Centre for Agricultural Research, Hungarian Academy of Sciences, Budapest, Hungary

**Keywords:** Polyandry, Multiple mating, Male quality, Infanticide avoidance, Scent marking, Sexual conflict

## Abstract

Females often show multi-male mating (MMM), but the adaptive functions are unclear. We tested whether female house mice (*Mus musculus musculus*) show MMM when they can choose their mates without male coercion. We released 32 females into separate enclosures where they could choose to mate with two neighboring males that were restricted to their own territories. We also tested whether females increase MMM when the available males appeared unable to exclude intruders from their territories. To manipulate territorial intrusion, we introduced scent-marked tiles from the neighboring males into males' territories, or we rearranged tiles within males' own territories as a control. Each female was tested in treatment and control conditions and we conducted paternity analyses on the 57 litters produced. We found that 46 % of litters were multiply sired, indicating that multiple paternity is common when females can choose their mates. Intrusion did not increase multiple paternity, though multiple paternity was significantly greater in the first trial when the males were virgins compared to the second trial. Since virgin male mice are highly infanticidal, this finding is consistent with the infanticide avoidance hypothesis. We also found that multiple paternity was higher when competing males showed small differences in their amount of scent marking, suggesting that females reduce MMM when they can detect differences in males' quality. Finally, multiple paternity was associated with increased litter size but only in the intrusion treatment, which suggests that the effect of multiple paternity on offspring number is dependent on male–male interactions.

## Introduction

The adaptive significance of multi-male mating (MMM) or polyandry is unclear and controversial (Jennions and Petrie 2000; Hosken and Stockley 2003; Simmons 2005; Gowaty 2012). Unlike males, females are not expected to increase their reproductive success by mating with multiple individuals (Bateman 1948; Trivers 1972). Moreover, polyandry can incur a number of costs for females, in terms of time and energy expenditure (Daly 1978), elevated risks of predation (Rowe 1994), injuries (Siva-Jothy 2006), and sexually transmitted diseases (Magnhagen 1991), suggesting that there are compensating benefits for females. Several nonmutually exclusive hypotheses have been proposed to explain how females can potentially gain fitness benefits from polyandry (Jennions and Petrie 2000; Simmons 2005). MMM could provide females with direct benefits, such as parental care, nuptial gifts, or other resources from males (Arnqvist and Nilsson 2000; Hosken and Stockley 2003). In nonresource-based mating systems, polyandry might function to increase females' fertility (fertility assurance hypothesis) (Hoogland 1998) or to obtain a variety of indirect, genetic benefits for offspring (Simmons 2005), such as eliciting sperm competition to gain “good genes” (Kempenaers et al. 1992), increasing genetic compatibility of maternal and paternal genomes (Zeh and Zeh 1997), including inbreeding avoidance (Tregenza and Wedell 2002), and increasing offspring genetic diversity (Yasui 1998, 2001). Most studies on polyandry have focused on birds and insects, whereas relatively little attention has been paid to mammals (Clutton-Brock and McAuliffe 2009) or sexual conflict hypotheses (Arnqvist and Rowe 2005). Sexual conflict hypotheses suggest that polyandry may be due to sexual coercion, so that females obtain no benefits (Wolff 1985; Smuts and Smuts 1993; Clutton-Brock and Parker 1995), or it may function as an alternative mating tactic (Taborsky et al. 2008) to reduce sexual harassment (convenience polyandry) (Thornhill and Alcock 1983) or infanticide (infanticide avoidance hypothesis) (Hrdy 1979; Agrell et al. 1998). Infanticide is the main cause of offspring mortality in many mammals, and polyandry has evolved more often in mammal species whose young are vulnerable to infanticide (Wolff and Macdonald 2004). However, there are surprisingly few experimental tests of whether polyandry functions to reduce infanticide or sexual coercion in any species. Here, we conducted a study with wild-derived house mice (*Mus musculus musculus*) to test whether females show MMM when they can choose their mates and are not constrained by sexual coercion, and whether multiple paternity affects offspring number or size when females can select their own mates.

Male house mice are territorial, and females usually mate with the dominant, territorial male, though sometimes females also mate with neighboring territorial males (Oakeshott 1974; Bronson 1979; Potts et al. 1991; Montero et al. 2013). Surveys in wild populations of house mice (*Mus domesticus*) have found that 6 to 43 % (mean 25 %) of litters are multiply sired (Dean et al. 2006; Firman and Simmons 2008a). It is unclear why there is so much variation in multiple paternity among wild populations. This variation may be due to changes within females' MMM (conditional mating tactic) or differences between females (heritable or nonheritable personality trait) (McFarlane et al. 2011). It could also be due to differences in social or ecological conditions, as multiple paternity has been found to be correlated with population density (Dean et al. 2006; but see Firman and Simmons 2008a). Females may increase MMM under high density because dominant males can no longer defend their territories from intruders (Anderson 1961), and since females have less protection, they likely face more sexual coercion and risk of infanticide (Calhoun 1962; Ebensperger 1998). Infanticide is very common in mice (Huck et al. 1982; Elwood and Ostermeyer 1984; Manning et al. 1995), and several studies suggest that males kill pups that are not likely to be their offspring. For example, virgin males are highly infanticidal (Labov 1980; Huck et al. 1982; vom Saal and Howard 1982; Elwood and Ostermeyer 1984; Elwood 1985), whereas copulation reduces infanticidal behavior (Soroker and Terkel 1988). Territorial males kill pups outside their own territory, and nonterritorial males commit infanticide when they have not sired any offspring (Manning et al. 1995). Although it is often suggested, it is not known whether female house mice show more MMM when they encounter strange or infanticidal males or whether MMM reduces their risk of infanticide. A study on bank voles (*Myodes glareolus*) examined the consequences of monogamy versus polyandry and found that offspring of socially polyandrous females had higher survival than offspring from socially monandrous females (all litters were genetically polyandrous) (Klemme and Ylönen 2010). This finding supports the infanticide avoidance hypothesis; however, to explain the variation in multiple paternity, studies are also needed to test whether females are more likely to mate multiply when they perceive a higher risk of infanticide from males.

MMM has been shown to provide several indirect, genetic benefits in female house mice. First, females have increased mean pup survival when they mate with three different males within one estrus cycle compared to females that mate three times with the same male, indicating that polyandry increases offspring viability (Firman and Simmons 2008c). Second, paternity is biased towards nonsiblings when a female mates with both a sibling and a nonsibling, indicating that polyandry facilitates inbreeding avoidance and enhances the genetic compatibility (Firman and Simmons 2008b). Third, female house mice from polyandrous selection lines (16 generations) have increased reproductive benefits compared to females from monandrous selection lines, as their sons achieve higher reproductive success under natural conditions (Firman 2011). However, in all these studies, matings were arranged and it is not known whether multiple paternity provides indirect, genetic benefits when females are able to select their own mates—though the benefits may be even greater compared to when females are forced to mate with randomly selected males in terms of quality. Females show preferences for males of high quality (Ilmonen et al. 2009), and female mate preferences can provide indirect benefits (Drickamer et al. 2000), but it is unclear how variation in male quality affects female MMM or the consequences of multiple paternity. Male mice scent mark their territories and countermark the marks of intruding males (Gosling 1982; Hurst 1990), and females use males' scent marks to recognize territorial males (Drickamer 1992) and to assess males' competitive ability (i.e., males' ability to exclude intruders) (Rich and Hurst 1998; Rich and Hurst 1999). Females may prefer to mate with competitive, territorial males to reduce their risk of infanticide (“pup defense hypothesis”) (Ebensperger 1998), as well as obtaining indirect benefits. Female mice also use male scent marking to assess other aspects of male quality, including health (Zala et al. 2004) and genetic disease resistance (Zala et al. 2008a), and females may not show MMM when they can detect differences in the quality of the available males. Also, females may be more likely to engage in extra-pair matings when they have a poor quality mate (“trade- up hypothesis”) (Kempenaers et al. 1992). Thus, previous studies indicate that female mice can obtain indirect, genetic benefits by MMM, but studies are still needed to determine whether MMM is influenced by variation in male quality (or females' perception of male quality) and how multiple paternity affects offspring fitness when females can select their mates.

In our study, we allowed female mice (wild-derived *M. musculus musculus*) to choose to mate with either one or two neighboring males, which both had their own territory but were restricted from leaving it, and we conduced genetic paternity analyses to determine whether females produce single- or multiple-sired litters (we assume that multiple-sired litters were more likely to be the result of MMM than single-sired litters). We also aimed to test whether females show more MMM, estimated by multiple paternity, when males are unable to defend their territories and exclude intruders, as occurs in high population densities. To test this hypothesis, we experimentally exchanged scent marks between the neighboring males' territories to simulate intrusion and manipulated males' apparent ability to exclude intruders (territorial intrusion). For controls, we relocated males' scent marks within their own territories. The experimental manipulation may alter females' perception of males' quality (in particular, their ability to defend their territory) and apparent risk of infanticide. We expected that if females use intruders' marks to assess males' ability to defend their territories and the risk of infanticide, then females should be more likely to have multiple-sired litters when males are unable to prevent intrusion. We also quantified male scent marking (as a measure of males' quality) to test whether differences in male quality affected the rate of multiple paternity. As only few studies have investigated the consistency or repeatability of females' MMM (Dietrich et al. 2004; Whittingham et al. 2006), we tested each female under territorial intrusion and control conditions. Finally, we examined whether multiple paternity resulted in increased number of offspring when females could select their own mates, as predicted by the fertility assurance, the intrinsic male quality, and the genetic compatibility hypotheses.

## Methods

### Experimental animals and housing

All experimental animals were F1 from wild-derived house mice (*M. musculus musculus*), which were trapped at 14 locations within a 500-m radius in Vienna (48°12′38″ N; 16°16′54″ E) and crossed between sites. The resulting F1 mice were weaned at the age of 21 ± 1 days and were thereafter housed individually in standard mouse cages (type II cages, 26.5 × 20.5 × 14 cm) under standard conditions (12:12 h light cycle) until the experiment was conducted. All cages were equipped with wooden bedding (ABEDD), wood shavings, and food (Altromin rodent diet 1324) and water ad libitum. We conducted ear punches for individual identification, and tissues were collected and stored at −20 °C for subsequent genetic analyses. Animals were between 3 and 5 months old when the experiment began. Mice were released into seminatural enclosures where the experiment was conducted.

### Experimental mate choice assay

**Fig. 1:**
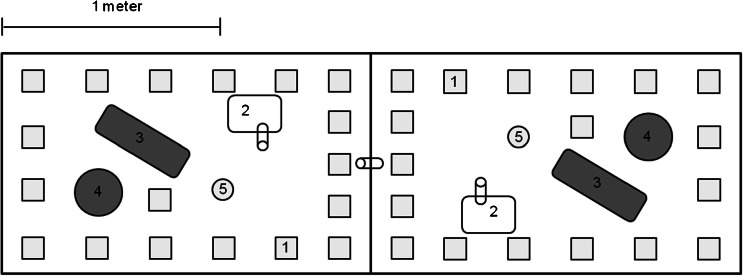
Neighboring males' compartments with a connection tube that allowed females to move between the male territories. Both compartments contained 18 tiles (*1*), a cage accessible only to females (*2*), a shelter box (*3*), a nest box (*4*), and a water dispenser (*5*). In the intrusion treatment, all the tiles in the two males' compartment were exchanged with each other, whereas in the controls, the tiles were rearranged within the males' own compartments

Each female (*N* = 32) could choose to mate with either one or both of two males (*N* = 64), which were located in two neighboring territories. Each territory (1 × 1.7 × 0.8 m) contained one nest box, one shelter, one mouse cage, one water dispenser, food (Altromin rodent diet 1324), and nesting material (Fig. 1). The males' enclosures were separated from each other by an opaque plastic divider to prevent them from entering and marking each other's territories. The divider had four holes (4 cm in diameter) at the base, which were mesh-sealed to allow visual and olfactory contact between males. Females could move freely between the males' territories through a small passage (plastic tube installed at the bottom of the divider, 3 cm diameter), whereas the males were prevented from entering the passage by collars (2.5-mm-wide cable ties with two attached wires that provided a mechanical barrier at the opening of the tube). Males were collared 2 days prior to their introduction to provide them with sufficient time to become habituated to the collar. A separate shelter cage was placed in each male's territory, which was only accessible to females through a narrow tube entrance that allowed females to escape sexual harassment.

### Scent marks and simulated intrusion

To collect scent marks, we placed 18 PVC tiles (10 × 10 cm) on the floor of each male's enclosures along the borders and next to nesting sites, covering approximately 11 % of the enclosure's surface area (Fig. 1). Each tile was individually labeled underneath and was assigned to an exact position within the enclosure. Males were introduced into the enclosures 2 days before the females to enable them to scent mark their compartment and to establish a territory. Simultaneously to male introduction, 20 μl of female urine (pool of seven females collected on five consecutive days) were deposited between the nest box and the shelter as female urine has been shown to stimulate males to increase scent marking (Zala et al. 2008b). In the experimental intrusion treatment (*N* = 32 females), the males' tiles were exchanged with those of their neighbor's compartment (to simulate intrusion), while in the controls (*N* = 32 females), the males' tiles were collected and replaced within their own compartment. Tile shifting began at day 3 of the experiment, shortly before females were released into the experiment, and was conducted on a daily bases around noon, when animals were inactive. To prevent spreading scent among the tiles and enclosures, observers wore one-way plastic shoe covers and tiles were handled with one-way latex gloves. We took photographs of all tiles before relocating them to assess the amount of males' scent marking for 6 days starting at the day of female introduction.

### Experimental design

Each of the 32 females was used in two trials, once in the intrusion treatment and once with a different pair of males under control conditions (within-subject design). The time between the first and the second trial was 2 months. The order of treatments versus control was determined by applying stratified randomization to avoid sequential effects. Due to space limitations, we could not test all 32 females simultaneously, so we ran two groups per trial, where we tested 16 females and 32 males each. The number of treatment and controls was balanced within groups. The 64 males were also tested twice; however, males were assigned to new pairs for the second trial. We ensured that none of the experimental animals were familiar with or related to one another and male pairs were body-mass-matched within 0.5 g. Male body mass was measured shortly before they received their collar, and female body mass was measured at the day we released them into the experiment. We determined the differences between female and male body mass by calculating the mean body mass of male pairs and subtracting female body mass. All females had given birth to one litter before this experiment to control for potential order effects due to comparing virgin versus nonvirgin females. The males were all virgins on the first trial and at least 61 % were sexually experienced in the second trial (61 % of males sired offspring, but the number of males that mated could be higher). The mice in the experiment were allowed to interact for 18 days and then all animals were returned to the colony. Males' collars were removed immediately and females were placed individually in type IIL mouse cages (32 × 20.5 × 14 cm) to give birth under controlled conditions. Reproductive success (litter size at birth and mean pup body mass at weaning [litter mass at weaning / litter size at weaning]) was measured, and genetic paternity analyses were conducted.

### Scent mark analysis

Photographs of tiles were recorded in a black box (60 × 60 × 80 cm) under UV light, emitted by two 18 W strip lights (90 cm, OMNILUX) fixed on the ceiling of the box. The 18 tiles within each territory were photographed in two sets of nine tiles each. Tiles were placed centrally on the bottom of the box in the same order and the same position. Digital photographs were recorded with a camera (Canon EOS 400 D Digital camera, 0.8 in. exposure time and 4.5 aperture value) from a fixed position on top of the box. We recorded the photographs of the first group in trial 1 (see “Experimental design” Section) in JPG format, but we excluded these data from our analyses as the image quality was inadequate and we only analyzed subsequent photographs which were recorded in CR2 format. The box was cleaned with 70 % ethanol after each photo to prevent odor contamination. Photographs were imported into Adobe Photoshop CS5.1 for image analyses and interpolated (10 cm ≙ 1,000 pixel) before a threshold was assigned. To assess the amount of individual male's scent marking, we determined the proportion of freshly marked tile area each day and calculated the sum of the freshly marked area over time starting after female introduction (sum of 5 days). For further analyses, we calculated the *difference* in the sum of marked tile area within male pairs (hereafter “*difference* in the two males' scent marking”). In addition, we determined the measurement error in taking three photographs of the same set of tiles and analyzed the photographs with the aforementioned method. The marked tile area differed in <0.05 % between the three photographs.

### Genetic paternity analyses

We conducted paternity analyses to define single- versus multiple-sired litters. DNA was extracted from ear punch samples using a proteinase K/isopropanol protocol (Sambrook et al. 1989). Individuals were genotyped at a minimum of six polymorphic microsatellite loci. If paternity could not be assigned by complete exclusion, we genotyped additional loci. A maximum of 16 microsatellite loci was used for paternity analyses (D11Mit150, D9Mit34, D9Mit135, D17Saha, D17Mit28, D10Mit20, D2Mit252, D6Mit138, D15Mit16, D5Mit25, D19Mit39, D7Mit227, D1Mit456, D2Mit380, D17Mit21, D1Mit404, see Mouse Microsatellite Data Base of Japan) using a Multiplex PCR MasterMix (Qiagen Multiplex PCR kit). Amplification mixes were subjected to a denaturation step at 94 °C for 15 min followed by 30 cycles at 94 °C for 30 s, 55 °C for 90 s, and 72 °C for 60 s, followed by an elongation step at 72 °C for 10 min. Amplification products were analyzed using an automated sequencer (Beckman Coulter CEQ 800). Allele scoring was performed using Beckman Coulter CEQ 8000 System software, and allele sizes were determined with SLS+400 as size standard. Paternity assignment was assessed using complete exclusion. In addition, paternity results were confirmed with a 95 to 99 % trio confidence (dam–sire–offspring relationship) using the program CERVUS 3.0.3 (Kalinowski et al. 2007).

### Statistical analyses

To test the effect of intrusion treatment and male scent marking on the rate of multiple-sired litters, we ran a generalized linear mixed effects model (GLMM) with a binomial error distribution and a logit link function. We entered paternity (single or multiple) as the dependent variable, trial and treatment as fixed effects, and female body mass, the body mass difference between males and females, and the *difference* in the two males' scent marking as covariates. As females were repeatedly tested, we included female ID as a random factor to control for nonindependence. Fitness effects of multiple mating were analyzed using a general linear mixed effects model (LMM) with either litter size or mean pup body mass as dependent variables, trial, treatment, and paternity as fixed effects, and female body mass as a covariate. Female ID was again included as a random factor. To test for the relationship between mean pup body mass and litter size under intrusion, we ran a linear model (LM) with mean pup body mass as the dependent variable and litter size as a covariate. Female ID was not included as a random factor as each female was tested only once under intrusion. We tested whether model assumptions (i.e., normally distributed residuals and homogeneity of variances) were fulfilled and transformed data if necessary. We only included biologically meaningful two-way interactions into all initial models and applied a backward stepwise removal procedure (Grafen and Hails 2002) to avoid problems due to inclusion of nonsignificant terms (Engqvist 2005). Removed variables were reentered one by one to the final model to obtain relevant statistics. Statistical analyses were performed using “R” (version 2.14.1). We implemented linear mixed effects models using the “lme” function of the “nlme” package, and generalized mixed effects models using the “lmer” function in the “lme4” package (R Development Core Team 2011).

## Results

**Fig. 2:**
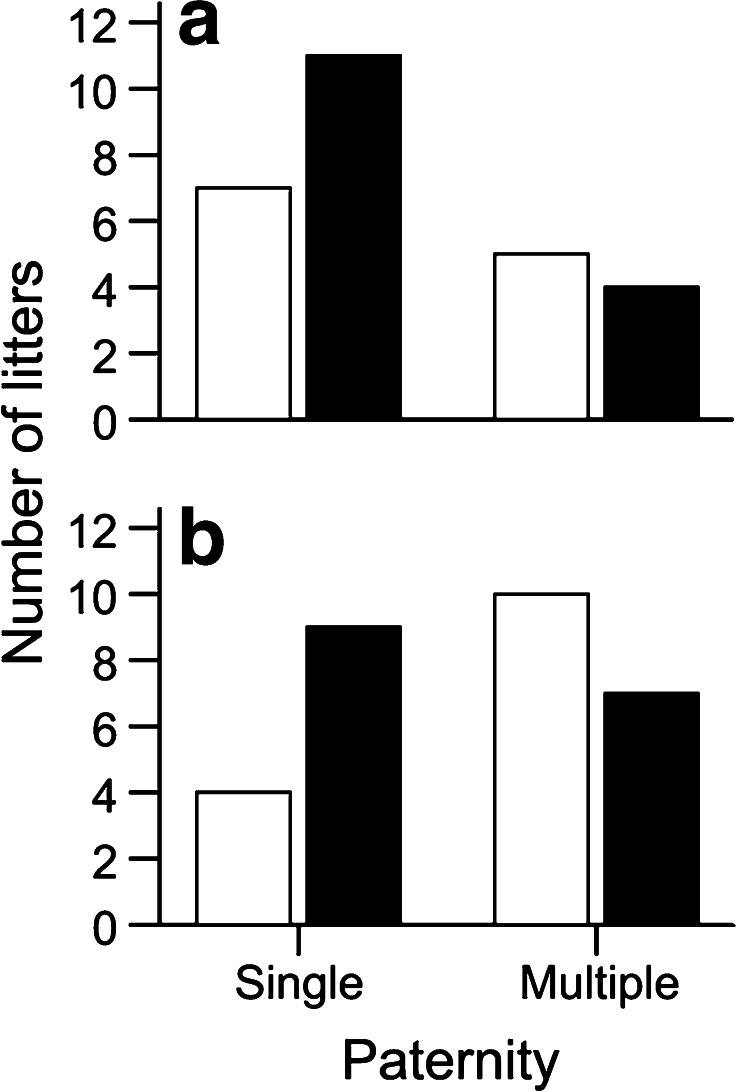
Frequency of single- and multiple-sired litters in the first (*white bars*) and second (*black bars*) trial of the experiment under (**a**) intrusion treatment and (**b**) control conditions

**Fig. 3:**
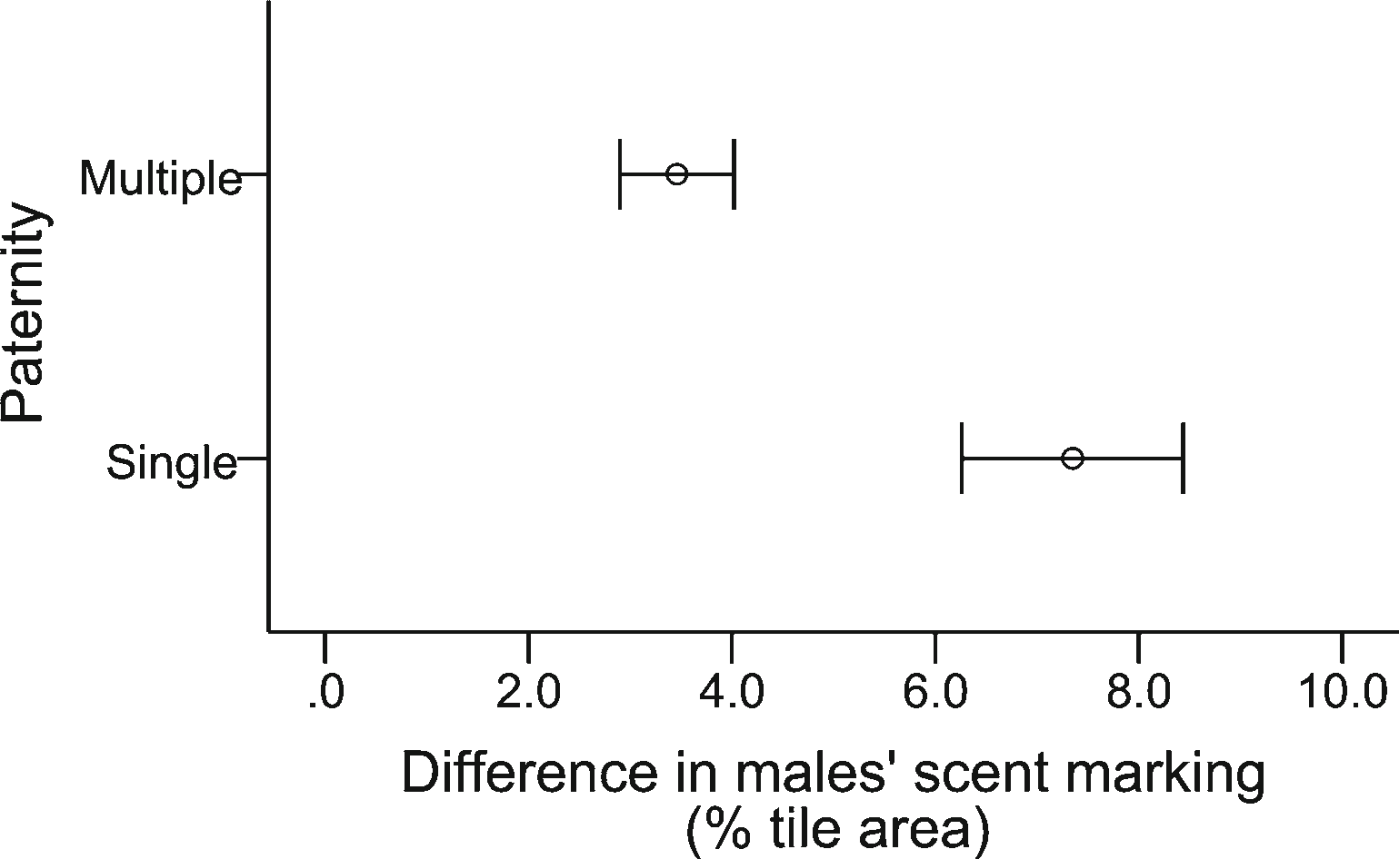
Difference in the two males' scent marking in single- and multiple-sired litters. Means ± SE are indicated

We found that 26 of the 57 litters (46 %) had multiple sires; however, the intrusion treatment did not significantly affect multiple paternity (GLMM, *z* = −0.283, *N* = 44, *P* = 0.777, Fig. 2). Yet, the rate of multiple paternity was significantly greater in the first trial when the males were all virgins (15/26 or 58 % of litters) compared to the second trial (11/31 or 35 % of litters) (GLMM, *z* = −2.306, *N* = 44, *P* = 0.021, Fig. 2). Also, paternity was significantly predicted by the *difference* in the two males' scent marking: we found that multiple paternity was higher when males showed smaller differences in their marking whereas single paternity was higher when the differences in males marking increased (GLMM, *z* = −2.472, *β* = −0.373, *SE* = 0.151, *N* = 44, *P* = 0.013, Fig. 3). We found no evidence that female body mass (GLMM, *z* = 0.911, *N* = 44, *P* = 0.362) or the differences in body mass between females and males (GLMM, *z* = −0.198, *N* = 44, *P* = 0.843) affected multiple paternity.

**Fig. 4:**
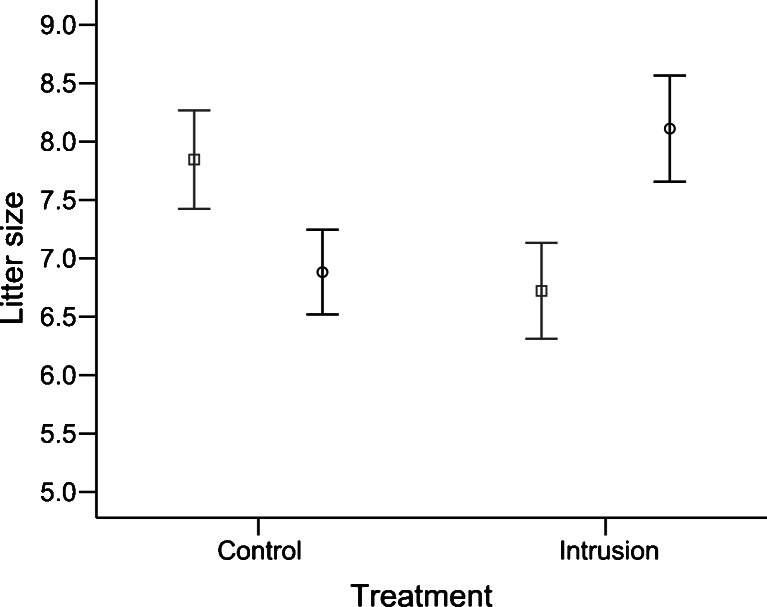
Litter size of single- (*gray*, *square*) and multiple- (*black*, *circle*) sired litters under intrusion treatment (single paternity, 6.72 ± 0.4, *N* = 18; multiple paternity, 8.11 ± 0.5, *N* = 9) and control conditions (single paternity, 7.85 ± 0.4, *N* = 13; multiple paternity, 6.94 ± 0.4, *N* = 17). Means ± SE are indicated

We found no overall effect of multiple paternity on litter size (LMM, *F*_1, 20_ = 2.861, *N* = 57, *P* = 0.106), but we found a significant interaction such that the effect of paternity on females' litter size was dependent on the experimental treatment (LMM, *F*_1, 20_ = 4. 671, *N* = 57, *P* = 0.043, Fig. 4). We therefore examined the effect of paternity independent for the intrusion treatment and the controls. In the intrusion treatment, litter size was significantly larger in multiple than single-sired litters (*T* test, *t* = −2.267, *N* = 27, *P* = 0.034) whereas paternity had no significant effect on litter size under control conditions (*T* test, *t* = 1.677, *N* = 30, *P* = 0.106). There was no change in litter size over trials (mean ± SD, 7.26 ± 1.60) (LMM, *F*_1, 19_ = 0.537, *N* = 57, *P* = 0.473), but heavier females had larger litters (LMM, *F*_1, 20_ = 6.925, *β* = 0.258, *SE* = 0.098, *N* = 57, *P* = 0.016). Female body mass did not predict mean pup body mass (LMM, *F*_1, 18_ = 0.822, *N* = 56, *P* = 0.376), though mean pup body mass significantly increased from the first to the second trial (from 8.57 g to 9.32 g) (LMM, *F*_1, 19_ = 12.686, *N* = 56, *P* = 0.002). The interaction between treatment and paternity also had a significant effect on mean pup body mass (LMM, *F*_1, 19_ = 7.739, *N* = 56, *P* = 0.012). We therefore, again, examined the effect of paternity independent for the intrusion treatment and the controls. In the intrusion treatment, mean pup body mass was significantly smaller in multiple-sired litters (*T* test, *t* = 3.391, *N* = 27, *P* = 0.002) whereas in the control treatment we did not find an effect of paternity on mean pup body mass (*T* test, *t* = −0.529, *N* = 29, *P* = 0.601). Thus, under intrusion, litter size increased whereas mean pup body mass decreased with multiple paternity. This result could be explained by the marginally nonsignificant negative relationship of litter size and mean pup body mass (LM, *F*_1, 25_ = 3.634, *β* = −0.264, *SE* = 0.131, *N* = 27, *P* = 0.068).

## Discussion

In total, 46 % (26 out of 57) of the litters were multiply sired, which is in the high end of the range of multiple paternity found in wild populations of house mice (Dean et al. 2006; Firman and Simmons 2008a). Thus, our findings show that females actively mate multiply when they have the opportunity to freely choose their mates and that they even increase MMM when they are unconstrained by males or other factors in the wild. A previous study on house mice (using offspring from crosses of female laboratory mice (ICR) with wild male mice (*Mus musculus domesticus*)) observed that 95 % of females actively mated multiply when they could choose to mate with a dominant versus a subordinate male (Rolland et al. 2003). Taken together, these findings support the hypothesis that MMM is due to female choice in house mice, whereas they are inconsistent with the hypothesis that females are forced to mate multiply (sexual coercion) (Wolff 1985; Smuts and Smuts 1993; Clutton-Brock and Parker 1995). We did not explicitly test consistency in MMM (for review, see Nakagawa and Schielzeth 2010) but since multiple paternity was inconsistent across trials, our findings do not provide evidence for consistent individual variation (personality trait or true alternative mating strategy) in multiple paternity. Although multiple paternity was not consistent between treatment versus controls, we found no evidence that multiple paternity was increased in the intrusion treatment, as predicted by the infanticide avoidance hypothesis. Females may have failed to detect any differences between intrusion versus control conditions, though this seems unlikely as the males scent marked significantly more under intrusion compared to control conditions (Thonhauser et al. submitted). Alternatively, females may not assess males' competitive ability based on the frequency of intruders' scent marks, as previously suggested (Rich and Hurst 1998), or males' competitive ability is unrelated to the risk of infanticide, contrary to the “pup defense” hypothesis (Ebensperger 1998). Thus, we cannot exclude the idea that multiple paternity depends on the risk of infanticide or males' perceived quality, especially since we found evidence for both hypotheses, as we explain below.

We found that the rate of multiple paternity was significantly higher in the first compared to the second trial, and females likely faced a higher risk of infanticide during this time. In the first trial, the available males were all still virgins—which are highly infanticidal (Labov 1980; Huck et al. 1982; vom Saal and Howard 1982; Elwood and Ostermeyer 1984; Elwood 1985). After copulation and cohabitation with a female, males reduce infanticidal behavior towards their mates' offspring and even other females' pups (Soroker and Terkel 1988). Thus, our finding suggests that female mice mate with multiple males when they encounter virgin males, as predicted by the infanticide avoidance hypothesis. It is not known whether female mice can recognize virgin or other infanticidal males, but females might discriminate differences in their scent, ultrasonic vocalizations, aggression, or other behaviors. Alternatively, the reduction in multiple paternity we observed may be due to other changes in males' behavior over time, unrelated to infanticidal behavior, or changes in females' behavior (despite that experimental females were nonvirgins in both trials). Experience might allow females to avoid male harassment and coercion, though this explanation seems unlikely, as females had a refuge and the differences in body mass between females and males had no influence on the multiple paternity rates. Alternatively, experience may allow females to more effectively defend their offspring against infanticidal males.

We measured males' scent marking since this behavior is expected to influence females' mating preferences, and indeed we found that the *difference* in the two males' scent marking explained the variation in single versus multiple paternity: in single-sired litters, the males displayed significantly larger differences in their scent marking compared to multiple-sired litters. Males' scent marking is a quality indicator display, and female mice can assess several aspects of quality on the basis of males' scent marking, including social status (Drickamer 1992), competitive ability (Rich and Hurst 1998; Rich and Hurst 1999), and health (Zala et al. 2004). Therefore, this finding suggests that females mate singly when they can detect differences in the males' quality and otherwise they mate multiply. There are several (nonexclusive) hypotheses to explain why females might use such a strategy. First, when females cannot detect differences in males' quality, they may mate multiply to incite sperm competition to increase the genetic quality of their offspring. This idea assumes that males' sperm competitiveness and offspring fitness are genetically correlated and males of high genetic quality sire more viable offspring (intrinsic male quality hypothesis) (Yasui 1997; García-González and Simmons 2005). Second, if male sperm competitiveness is heritable, multiply mated females would have a selective advantage over single mated females as the former are fertilized by the most competitive sperm and will have sons which have superior sperm competitive abilities. A study on house mice showed that polyandrous females can gain fitness benefits by producing sons that achieve high reproductive success in a competitive environment (Firman 2011). Third, if females cannot detect differences in males' quality, MMM could provide females with good genes as females avoid sampling errors caused by inadequate mate discrimination (bet-hedging) (Yasui 1998). Fourth, this result may not be due to female choice, but rather to male sperm competitiveness and male–male interactions. Females may have generally mated multiply, but males' scent marking might have honestly reflected male sperm competitiveness and males that marked at similar rates were equally good in sperm competition, or alternatively, higher marking males could have been better in intimidating rivals, which then in turn transferred less sperm. Future studies are needed that include direct observations of female and male behavior to determine whether our results can be explained by female choice, male–male competition, or an interaction of both.

Finally, we aimed to determine whether multiple paternity enhanced females' reproductive success (litter size) when they are able to select their mates. We found no overall effect of multiple paternity on litter size, but we found an unexpected interaction that masked the effect of paternity on litter size: multiple paternity increased litter size in the intrusion treatment, whereas there was no significant effect in the controls. Thus, although intrusion treatment had no effect on multiple paternity, the effects of multiple paternity on offspring number crucially depended on intrusion where male–male interactions were intensified (and the within-subject design controls for other potential confounds). This finding suggests that multiple paternity provided reproductive (fitness) benefits for females (e.g., as predicted by fertility assurance, the genetic compatibility, and intrinsic male quality hypotheses), but why did this effect only occur under intrusion treatment? One possible explanation is that males perceived higher competition with intrusion and they increased the number of sperm transferred during mating when they perceive a high risk of sperm competition (Parker 1990, 1998; Wedell et al. 2002). One study on house mice supports this hypothesis (Ramm and Stockley 2009a), but a second study found no support (Ramm and Stockley 2009b) and a third study found that males reduced the number of sperm transferred when mating in the presence of a rival male (Ramm and Stockley 2007). Therefore, it is unclear whether males transfer more sperm when they perceive an increased risk of sperm competition. Moreover, it is unlikely that the females were sperm limited under intrusion but not under control conditions. Interpreting the positive correlation is not straightforward because increasing offspring number does not necessarily enhance females' fitness, contrary to what is often assumed, as there are sexual conflicts over the optimal number of offspring (Penn and Smith 2007), as well as over parental investment. If females engage in multiple mating to reduce the costs from sexual harassment or to reduce infanticide, then increases in litter size from MMM may be costly rather than beneficial for females' fitness. The situation becomes even more complicated when we consider that multiple paternity exacerbates sibling rivalry, as well as sex conflicts, and offspring can potentially influence maternal investment and litter size (Royle et al. 2004; Drake et al. 2008). Males and their offspring may influence the number of eggs that females produce or the number of embryos reabsorbed (Hager and Johnstone 2003). An increasing number of studies find that male–male competition influences female mate choice (Wong and Candolin 2005), but this is the first study to our knowledge that shows that the effects of multiple paternity on offspring number depend on females' exposure to male–male interactions. Thus, future studies are needed to determine why the effects of multiple paternity on offspring number depend on male–male interactions and to disentangle the underlying proximate causes and the evolutionary fitness consequences.

Litter size was also influenced by maternal body mass with heavier females producing larger litters. Given that reproduction incurs fitness costs (Reznick 1985), and especially in mammals (Speakman 2008) where gestation is followed by lactation, females in better condition and larger body mass could probably better afford the costs of producing larger litters. Yet, mean pup body mass did not depend on female body mass. Instead, pup body mass significantly increased over trials, indicating that older or more experienced females invested more resources into their average offspring or that they reduce investment when exposed to infanticidal virgin males. Females were repeatedly tested and could gain experience in mate choice and the raising of pups. Although not all females became pregnant during the first trial, all of the females in our study were sexually experienced and gave birth to one litter before used in this experiment. This way we ensured that any differences in the litter sizes between the trials were not due to comparing virgin and nonvirgin females. Pup body mass at weaning is known to correlate with offspring survival in the wild (Baker and Fowler 1992). Our results thus suggest that offspring number depends on female body mass whereas offspring quality (e.g., mean pup body mass) depends on females' age or experience. We also found a negative relationship between litter size and mean pup body mass under intrusion, indicating a negative trade-off between offspring number and quality (Smith and Fretwell 1974).

In summary, we found high rates of multiple paternity even when females can select their mates, indicating that MMM is due to female choice rather than sexual coercion. We found no evidence that females were more likely to give birth to multiple-sired litters when males' territories were intruded by neighboring males, as expected if MMM functions to reduce infanticide. We found that multiple paternity was significantly increased in the first trial when the available males were virgins, which are known to be highly infanticidal. However, experimental tests are needed to determine whether MMM is increased when females are exposed to virgin or otherwise infanticidal males. Also, multiple paternity was influenced by the difference in the two males' scent marking, which is a condition-dependent secondary sexual trait (quality indicator). This finding suggests that females mate singly when they are able to detect significant differences in male quality and otherwise they mate multiply (e.g., bet-hedging hypothesis). Finally, we found that multiple-sired litters were larger than single-sired litters under intrusion, though studies are needed to determine why this effect only occurred under intrusion when male–male interactions were intensified. Future studies should be aware that the effects of multiple paternity on female reproduction (offspring number) can be masked and even reversed by male–male interactions.
